# First-line managers struggling to lead home care based on the individual’s needs and goals – conflict between ethical principles

**DOI:** 10.1108/LHS-05-2023-0035

**Published:** 2024-01-23

**Authors:** Inger James, Annica Kihlgren, Margaretha Norell Pejner, Sofia Tavemark

**Affiliations:** Faculty of Health, Science, and Technology, Karlstad University, Karlstad, Sweden and Research Environment: Older People’s Health and Living Condition, Örebro University, Örebro, Sweden; School of Health Sciences, Örebro University, Örebro, Sweden and Research Environment: Older People’s Health and Living Condition, Örebro University, Örebro, Sweden; School of Health Sciences, Örebro University, Örebro, Sweden; Department of Home Care, Halmstad Municipality, Halmstad, Sweden and Research Environment: Older People’s Health and Living Condition, Örebro University, Örebro, Sweden, and; Örebro Municipality Healthcare and Social Services, Örebro, Sweden and Research Environment: Older People’s Health and Living Condition, Örebro University, Örebro, Sweden

**Keywords:** Home care, Individual goal, Leadership, Participatory appreciative action reflection, Value-based leadership, Ethical principles, Organizational culture

## Abstract

**Purpose:**

The purpose of this paper is to describe how first-line managers (FLMs) in home care (HC) reason about the opportunities and obstacles to lead the work according to the individual’s needs and goals.

**Design/methodology/approach:**

In this participatory appreciative action reflection project, eight managers within one Swedish municipality were interviewed. The data were analysed using a thematic analysis.

**Findings:**

The results showed a polarization between two different systems that FLMs struggle to balance when attempting to lead HC that adapts to the needs and goals of individuals. One system was represented by the possibilities of a humane system, with human capital in the form of the individual, older persons and the co-workers in HC. The second system was represented by obstacles in the form of the economic needs of the organization in which the individual receiving HC often felt forgotten. In this system, the organization’s needs and goals governed, with FLMs needing to adapt to the cost-effectiveness principle and keep a balanced budget. The managers had to balance an ethical conflict of values between the human value and needs-solidarity principles, with that of the cost-effectiveness principle.

**Originality/value:**

The FLMs lack the opportunity to lead HC according to the needs and goals of the individuals receiving HC. There is a need for consensus and a value-based leadership model based on ethical principles such as the principles of human value and needs-solidarity to lead the HC according to the individual’s needs and goals.

## Introduction

Health managers in elderly care worldwide face complex challenges such as demographic and epidemiological changes, labour shortages, changing structures and efficiency savings (Figueroa *et al.*, 2019). Today, the principle prevails worldwide that older persons should have a healthy ageing in which each person is able to live and do what he or she values (WHO, 2021). However, as people age, diseases are more common (WHO, 2022), and people are living longer with complex health problems (Meinow *et al.*, 2022). Despite this they still should be able to grow old in their own home in what is called “ageing in place” (WHO, 2018) and receive health and social care (Jacobs, 2019) in the form of home health care.

In Sweden, the government and the Ministry of Social Affairs are responsible for the care of older people at the state level. Thereafter, it is the municipalities and the elected local politicians who have the ultimate responsibility for the care of older people. Elderly care in the municipalities can be run privately, but it is the municipality that has the uttermost responsibility (Hagerman *et al.*, 2019; SOU, 2017:21a). Home care (HC) is mainly tax financed, but the individual is charged various fees depending on their income (Tavemark *et al.*, 2023). The municipality is managed on several levels and the first-line manager (FLM) is the link between the management system and the staff in health-care organizations (Ericsson and Augustinsson, 2015; Guest and Woodrow, 2012). Furthermore, FLM is crucial to how practices are designed (Ericsson and Augustinsson, 2015; Solbakken *et al.*, 2019) where elderly care should be tailored in relation to the needs of the individual (Kihlgren *et al.*, 2023).

Responsibilities associated with the different positions in the managerial structure may vary, and the hierarchical levels may also vary with the various positions (SOU, 2017:21b). Leaders in elderly care are in a rigid organization, with the character of institutions (Kihlgren *et al.*, 2023) where different control systems are in charge which can prevent leaders from taking decisions (Hagerman *et al.*, 2019). It is generally accepted that HC should be based on a holistic view of the citizens, with the responsibility for implementation resting on “three legs”: the business, finance and personnel (SKL, 2011). FLMs are responsible for creating conditions for the co-workers providing the care to carry out their assignments with the desired quality for citizens. They also have financial responsibility for establishing the budget and being responsible for ensuring that the business is cost-effective. The FLMs have the responsibility of leading and distributing the work to prioritize between older individuals’ wishes, securing the work environment, clarifying expectations and providing feedback on results (National Board of Health and Welfare, 2021a).

Municipal HC is split between home health care and HC service. Registered nurses (RN) and occupational therapists (OT) offer the individuals home health care that is based on the Health and Medical Care Act (SFS, 2017:30). Nurse assistants (NA) and care aids (CA) offer HC service that is based on the Social Service Act (SFS, 2001:453). In the following text, HC will refer to both home care and home health care. It is stipulated via various governing documents that health and social care should be done in participation and collaboration with the individual (National Board of Health and Welfare, 2021b; SFS 2014:821; SFS 2017:30), based on national core values in which dignity, well-being and a meaningful daily life are central values (Kihlgren *et al.*, 2021; National Board of Health and Welfare, 2012). HC should be performed in a person-centred manner based on the individual’s goals (National Board of Health and Welfare, 2021b). The professions have different roles in HC. The co-workers who mainly perform the person-centred care are CA and NA who know the individuals and their needs best because they spend most time with the individual (Lidström Holmqvist and James, 2019). OT and RN are responsible for rehabilitation and nursing interventions according to individual’s needs and goals (Tavemark *et al.*, 2023) and delegate interventions to NAs. However, a gap exists between the individual’s rights according to guidelines and legalisation and the HC the individual receives (Jarling *et al.*, 2018). The gap also consists of deficiencies in medical care and in patient safety (Health and Social Care Inspectorate, 2022). Reasons of deficiencies in care can be a deprived work environment and insufficient resources (Andersson *et al.*, 2022).

The FLMs can affect the quality of HC (Johannessen *et al.*, 2021). However, leadership can be seen as complex (Lindberg *et al.*, 2022) in a complex everyday life (Ericsson and Augustinsson, 2015). FLMs describe the work as meaningful, especially when they lead the work, even though they consider their work demanding and onerous (Hagerman *et al.*, 2019). FLMs have an unpredictable job (Wastesson *et al.*, 2021) and a difficult work situation where they are intermediaries between upper level (top management) and local level (their co-workers) (Strömberg *et al.*, 2019). FLMs must deal with different values and experience ethical problems, such as difficulties in satisfying the individual’s need for autonomy and self-determination, and there is a lack of organizational conditions (Jonasson *et al.*, 2019) that makes it difficult to achieve the individual’s goals (National Board of Health and Welfare, 2021b). Structural changes are needed for the individual to receive the HC to which they are entitled (Lidström Holmqvist and James, 2019; Tavemark *et al.*, 2023) and pay for. In line with this, knowledge is needed about what opportunities and obstacles exist in HC to working according to the individual’s needs and goals (Tavemark *et al.*, 2023).

The purpose of this study is to describe how FLMs in HC reason about the opportunities and obstacles to lead the work according to the individual’s needs and goals.

## Material and methods

This study is part of a larger project examining a municipality in central Sweden that implemented a structural change programme to address individual needs and goals in HC. To learn from those who have experience of HC and to allow their voices to be heard, participant-based research was conducted. The participant-based research method is characterized by the stakeholders and the researchers collaborating to develop and improve the practice (Ghaye *et al.*, 2008; James *et al.*, 2015). A characteristic of the participatory appreciative action reflection method is that opportunities are seen and thereafter implemented in practice, and together the stakeholders seek solutions to obstacles (Ghaye *et al.*, 2008). In participatory research, the stakeholders effectively become co-researchers (James *et al.*, 2015).

### Study setting

This study occurred between 2019 and 2021, to develop working system based on the individual’s needs and goals. All HC units (*n* = 20) were supposed to participate; the top management selected three HC units to start with, depending on which units had capability for participating. Thereafter, another two. Because of the COVID-19 pandemic, additional departments were not recruited, and a total of five units participated.

In the project, a total of 160 stakeholders were included: older persons (65 years and older), their relatives, CAs, NAs, OTs, RNs, staff from the authority and FLMs in the HC teams. This study is based on the interviews of the FLMs from the included HC units, i.e. home health care and HC services. A total of nine FLMs were invited, and eight chose to participate in this study, two men and six women who have worked as FLMs for 1–25 years (see Table 1).

### Data collection

FLMs were asked to participate in initial and follow-up interviews (Kvale and Brinkmann, 2015). In total, 11 interviews were conducted with a focus on leading HC work based on the individual’s needs and goals. In line with participatory research where the intention is to involve the participants as co-researchers, the interviews were open and were carried out as a conversation. The two authors who conducted the interviews (IJ and ST) have extensive experience in interview technique. It was explained that the goal was to learn from the FLMs’ experience and knowledge. In the conversations, we focused on opportunities to lead the work according to the needs and goals of the individuals. We asked about obstacles and whether they had suggestions to solve the obstacles. The prerequisites needed and missing were discussed, and participants were asked to identify problematic situations. Furthermore, we asked whether the way of working can be changed in any way to work according to the individual’s needs and goals. Follow-up questions were asked such as: Can you elaborate? Can you give an example? In the follow-up interview, which was approximately two months after the first, we presented what the participants had said previously and asked whether they had thought of anything else? In this way, questions and reflections could be exchanged and the data could be confirmed. It was also a method to get deeper into the co-creation of knowledge.

All FLMs were individually interviewed face-to-face, except for two managers for whom a group interview was conducted via Zoom because of COVID-19. The interviews lasted 1–1.5 h, were digitally recorded and were transcribed verbatim.

### Data analysis

To report the FLMs’ perspective, a thematic analysis according to Braun and Clarke (2006) was chosen. The authors, (IJ and ST), read all the interviews to ensure credibly that the data was not disclosed. In a second step, we went through the content that answered the purpose and wrote down codes, where the codes were grouped by content distinguishing similarities and differences. All the codes were collected into preliminary themes in an initial map. Relationships between codes and different levels of themes were analysed. Furthermore, to ensure credibility, the authors (IJ and ST) refined the themes, where sub-themes and codes were compared with each other, and the whole, where the sub-themes gave structure to the main themes. The main themes illustrate the meaning of each sub-theme. To provide trustworthiness, all authors (IJ, AK, MNP and ST) strove to test the themes in relation to their labels and content and describe each theme accurately. Finally, quotes were used to illustrate the meaning of each theme, with feedback to the purpose to confirm the results.

### Ethical considerations

Ethical approval was granted by the Swedish Ethical Review Authority. Several ethical considerations were made in relation to the FLMs. The risk that the FLMs could feel that their work and professionalism was being questioned was managed with information both in writing and verbally and with written informed consent.

All FLMs received written and oral information and provided written informed consent. They were also informed that they could end their participation at any time without giving an explanation. All data material was coded and kept in a locked safe.

## Results

**Figure 1.**
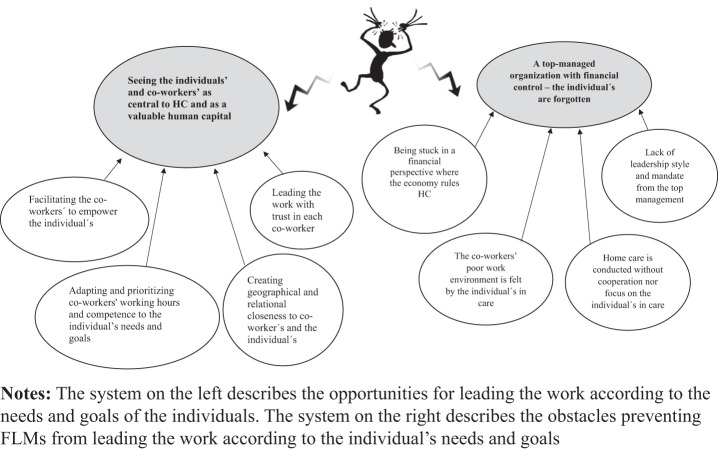
The polarization between two systems, represented by two main themes: “seeing the individuals and co-workers as central to HC and as a valuable human capital” and “a top-down managed organization with financial control – the individuals are forgotten”, each with four sub-themes, which FLMs in HC struggle to find a balance between

The results showed a polarization between a human system and a system of obstacles consisting of a top-down managed organization that FLMs struggle to balance when leading HC (Figure 1). One system occurs when the individuals and co-workers are central to FLMs, which is a humane system with human valuable capital in the form of the individuals and the co-workers. In this system, the FLMs can see opportunities in leading the work according to the individual’s needs and goals. The second system is represented by obstacles consisting of a top-down managed organization, which focuses on financial control, in which the individual can feel forgotten. In this system the FLMs are stuck in a top-down system and lack the mandate to lead the work.

## Seeing the individuals and co-workers as central to HC and as a valuable human capital

The first main theme describes a system that is characterized on the individual in the centre with a trust in co-workers. The FLMs stated that an opportunity for leading HC according to the individual’s needs and goals was to facilitate the co-workers to empower the individual. For example, by adjusting their working hours and competence needed to be adapted to the individual’s needs and goals. Another opportunity needed was to create geographical and relational closeness to co-workers and the individuals, as well as lead the work with trust in each co-worker to reach the individual’s needs and goals. The co-workers and the individuals need to be seen as valuable human capital.

### Facilitating the co-workers to empower the individuals

The FLMs asserted that an opportunity for being able to lead according to the individual’s needs and goals was if the whole organization “change their thinking” and have a consensus with the individual’s needs and goals at the centre instead of focusing on time and finances. The existence of HC depends on, as one FLM expressed, having “the individual […] should be the central person. Because I keep thinking that without them, we don’t exist”. One proposal for change was that the FLM must lead co-workers towards a common focus of reablement, as the individual’s goal can target achieving independence in daily life and by that facilitate the individual to feel empowered. The FLMs talked about the possible goals of older persons: “I want to be able to clean myself. I want to be more mobile or for my pressure sore on my backside to go away.”

Documentation of the individual care plan at home together with the individual was described as a suggestion to facilitate a sense of empowerment to the individual and to clarify the direction for HC. The FLMs said that the documented plan for HC should be described from the individual’s perspective regarding how the health and social care should be performed. Through documentation at home together with the individual, the older person could follow the documentation process and at the same time evaluate the HC. Another suggestion for change that was presented involved the older person doing the documentation themselves. The FLMs also suggested that digital solutions could make it possible for the individuals and their relatives, and the NAs (contact person) to participate in team meetings from the home, together with team members at the office.

### Adapting and prioritizing co-workers’ working hours and competence to the individual’s needs and goals

The FLMs discussed that an opportunity for leading the work according to the individual’s goals was to adapt the NA’s working hours, specifically the contact person’s, to the older person’s needs and goals. Another opportunity was for the team members with the most knowledge of the individuals to work the most closely with the individuals. Older persons with the most extensive needs and requiring several home visits during the day were considered to need the same co-workers to achieve a high degree of continuity. As one FLM described: “This person has the greatest need for continuity, with functional impairments, that you cannot handle impressions or if it is that you are seriously ill, then perhaps they are prioritized first, with closer continuity.”

The FLMs described opportunities such as working with mixed groups of co-workers with different experiences and training and who speak different languages. The person who needs palliative care should meet NAs with competence and training within palliative care. Older persons with dementia must meet NAs who are trained within those fields. Thus, the co-workers must receive continuous competence development in a variety of areas. Another opportunity was for the group to have a mix of both men and women, so that the older person does not have to receive help from someone of the opposite sex if they do not want it. This was described by a FLM as: “[…] but you might not want someone from another gender to shower you. We must be able to respect that. And then we have both women and men.”

### Creating geographical and relational closeness to co-workers and the individuals

The FLMs reasoned that an opportunity and a prerequisite for leading the work towards the individual’s needs and goals was to have their offices close to those of their co-workers and have an “open door” to be able to listen to the co-workers and to develop relations. The co-workers should feel that it is acceptable to come into the FLM’s office and talk, regardless of whether it is about work or home conditions. The FLMs emphasized that a relationship based on mutual trust is needed between the co-workers and the FLM because they depend on each other. The FLMs reasoned that closeness to the individuals, both geographically and relationally, was an opportunity and a prerequisite to allow for the individuals goals to be met. The NAs work most closely with the older person, have a close relationship and know the HC the individual wants. Also, the NAs can motivate and challenge the older person to reach his/her goals. The FLMs emphasized that a close relationship is most important, after which comes the practical tasks. The focus of the NA should be on the conversation and the responsiveness of the individuals. One FLM described how she would lead the work: “And I tend to be a bit provocative, I sort of say like this: think this way, you have been given two eyes and two ears and one mouth, and that means you should listen and observe twice as much as you talk.”

Relationships, cooperation and geographical proximity between different professions in the team is an opportunity described by the FLMs to lead the work by appreciating and making use of each other’s skills. Thus, smaller work groups and units with team members at the same place are needed. Then the co-workers can gain information and share knowledge about the older person, so that they can have a dialogue about how the individual’s needs and goals can be reached. One FLM described how distance affects cooperation: “I see differences in my area. Here in town we have two nursing groups. This one don’t have as good dialogue as the other one […].”

### Leading the work with trust in each co-worker

The FLMs thought that the co-workers were the prerequisite, an opportunity and their capital to lead the work according to the individual’s goals. Their work effort was seen as the HC’s resource and of great importance for the FLM and the individuals. One FLM stated: “My co-workers are very, very important to me. They are very important for the individuals.” The FLMs explained that they, as leaders, saw the positive in each co-worker and did not put energy into small mistakes. One approach of the FLMs was that they encouraged, confirmed good performance and were responsive to the co-workers’ ideas. The FLMs emphasized the co-workers’ engagement and said that they trusted and had confidence in them. One FLM praised them: “The most important thing a manager can do is to face his co-workers every day as if they were God’s gift to humanity.”

The FLMs discussed that an opportunity was a positive work environment that could cause the entire work group to treat each other well and thereby create the potential for a positive relationship with the older person, facilitating in meeting the individual’s needs and goals. The FLMs described their co-workers as loyal to each other and to the older persons. The mutual dependence in HC was seen as an opportunity to lead the work based on the individual’s needs and goals: “The individual is at the centre but without co-workers the individual cannot cope and the co-workers cannot cope without the individual.”

## A top-down managed organization with financial control – the individuals are forgotten

The second main theme describes another system that is characterized by the FLMs’ struggle to lead in a top-down managed organization focused on financial control in which there is a lack of consensus and the individual in HC can feel forgotten. Several obstacles to providing individual care in such a system are apparent, such as the FLMs being stuck in a financial perspective where the economy rules HC. Furthermore, the co-worker’s poor working environment affects the individual’s care environment. The organization is run without cooperation nor a focus on the individual in care. The FLMs describe that opportunities were lacking to implement their leadership, as they lack a mandate and dialogue from top management.

### Being stuck in a financial perspective where the economy rules home care

The FLMs stated that in a top-down system there are obstacles to lead the work according to the individual’s needs and goals. One obstacle is that the organization is constantly changing and reorganizing, with an economy that is not in balance. It was considered unrealistic to present changes to the co-workers and implement them in practice, as the financial conditions in the organization were lacking. One FLM explained: “For me to go out to my co-workers and talk about unrealistic goals that I myself feel are not realistic, there is no gain in that.”

The FLMs highlighted another obstacle that in a top-down system it is often politicians without experience in HC that govern the HC from above. At the same time, the FLMs have the financial responsibility for keeping the budget balanced. They discussed that there is a lack of consensus in how they as FLMs and the politicians and top managers value the co-workers and the individuals in HC. The FLMs described that the focus of top management is on the economy and that they only look at financial results. The FLMs felt that HC had evolved into a task orientated to keep costs down, where it is forgotten that HC is meant to work with humans. There is no opportunity to meet the individual’s needs and goals that may arise in the moment. One FLM stated: “So many times you see when a co-worker comes in and does the task and leaves, he or she doesn't look up and see the person and ask how they feel.”

The FLMs described time as an obstacle in that every minute at the older person’s home is counted as money. The FLMs considered that there is too little time calculated for the individual’s HC. Another obstacle described by the FLMs was the lack of sufficient financial compensation for the co-workers’ travel time to the older person and their time for documentation of their work.

### The co-workers’ poor work environment is the individual’s care environment

The FLMs expressed that sickness and absences of the co-workers were an obstacle to leading the work based on the individual’s needs and goals. The work environment was described as stressful because of the organization’s governance and the need for a balanced budget. It is hard both physically and mentally, especially for the NAs, leading to many co-workers being on sick leave, which costs money and negatively influences the remaining co-workers’ work environment. When the NAs are absent, the FLMs have to use substitutes who do not always know the individual and their needs and goals. Continuity of care suffers, and the relationship with the older person deteriorates, which reduces the individual’s care environment. One FLM described the conflict: “We can’t just think economics. Then we have co-workers who are not feeling well, the working environment is bad, and we get long-term sick leave.” The FLMs emphasized that the NAs could be the only work group in Sweden that do not get a coffee break and hardly get the opportunity to use the toilet during working hours. To solve this obstacle, the FLMs suggested that the work environment must be improved to reduce sick leave. This would improve the continuity of care and the care environment, meaning that the quality of care and the financial stability of the HC would also increase.

### Home care is conducted without cooperation nor focus on the individuals

The FLMs described that an opportunity needed to lead based on the individual’s needs and goals is cooperation between the hospital, primary care and HC, which is often lacking in a top-down system. For example, they experienced that the communication of patient needs between the hospital and the HC is often incomplete, sometimes resulting in the older person coming home from the hospital without the individual care plan setup and without the home environment being prepared. The FLMs described consequences such as increased workload and a poor work environment because HC does not have the resources for emergency interventions. For the individual, it becomes unsafe to come home, and their individual goals are not possible to reach. An FML described it by saying: “For example, medicine may be missing and then the nurse must solve it by delegating, if possible, or go and collect medicine herself.” The FLMs also highlighted another obstacle which is that different legislation often steer HC to be non-cooperative: “And there I have no solution, I send it further up in the organization for a decision.” This leads to the needs and goals of the individual not being met because the laws refer responsibility to each other.

### Lack of leadership style and mandate from the top management

The FLMs discussed problems related to the lack of clear leadership style adopted in the municipality. They get directives from above, and they must obey. It was described by one FLM as: “You run after everything.” The FLMs described an obstacle as a need to balance between the organization, the older person, relatives, the other professions in the team, the economy and the co-workers’ working environment. A proposal for change was to adopt a leadership style in HC to achieve results. The FLMs highlighted obstacles such as a lack of support and feedback from top management. They did not get feedback on whether they had done well or if things went badly. One FLM said: “There are many leads of top managers before we are reached or our co-workers if we even get any information or feedback from the politicians.” They also suggested that top management lacks the knowledge to guide according to the individual’s needs and goals, as they do not have health and social care training and do not understand how HC works at the individual level. The FLMs described that top management does not come to terms with the direction the HC should take. They described problems because of hierarchy and distance from top management to all employees. The information from top management about decisions and the purpose of changes does not reach all the way down to FLMs or to all co-workers. The FLMs said that neither they nor their co-workers were listened to by top management or were allowed to make suggestions or impact statements when changes were current. The FLMs described that further obstacles were that their hands were tied behind their back and that they lacked a mandate to deal with problems such as personnel issues. For example, when their co-workers behaved badly towards an older person, a written warning might be needed, but it is not something that the FLMs have a mandate to do.

## Discussion

The results showed a polarization of two different systems between which the FLMs struggle to balance. One system is represented by opportunities. The first main theme describes a system where the individuals receiving HC is in centre, with trust in co-workers. It is a humane system with human valuable capital in the form of the individuals and the co-workers. The second system is represented by obstacles through a financial control system in which the individuals can feel forgotten, a situation beyond the control of the FLMs. This struggle can be viewed as the FLMs being in an ethical value conflict. It is known that a struggle can arise when the individual is prevented from being present and involved in HC and is hindered by the organization’s demands and expectations (Blomberg *et al.*, 2019; Jonasson *et al.*, 2019; Solbakken *et al.*, 2021). Furthermore, the FLMs must balance between the human value and needs-solidarity principles and the cost-effectiveness principle, which can mean that there will be difficulties in leading the work according to the individual’s needs and goals. The human value principle means that all people have equal value regardless of, for example, age. The needs-solidarity principle means that those with the greatest need must be prioritized. According to the cost-effectiveness principle, there must be a reasonable balance between cost and efficiency where improved health and quality of life must be pursued. In line with the Swedish ethical platform for prioritization and fair distribution of resources, the cost-effectiveness principle must be subordinate to the human value and the needs-solidarity principles (Sandman and Kjellström, 2018; SOU14, 1996/97). The FLMs find themselves in a conflict of values in a top-down system dominated by the cost-effectiveness principle. They struggle to lead the work according to the individual’s needs and goals and to achieve financial results.

The FLMs saw opportunities and proposed changes to lead according to the individual’s needs and goals at work. One way was to lead the co-workers to focus on reablement which could facilitate the individual to feel empowered. This is accomplished through the individual’s plan for HC being documented from the individual’s perspective together at the individual’s home, and if possible involved the older persons doing the documentation himself/herself. In this way, the power of HC is reduced, and team collaboration increases, while the older person’s dignity and self-determination are promoted (Sundström *et al.*, 2017). Another opportunity and suggestion for change was to have digital team meetings at the older person’s home. Using digital systems, which is already done (SOU 2017: 21b), especially in sparsely populated areas can facilitate the individual’s empowerment, in terms of their ability to control their HC.

Furthermore, FLMs described an opportunity to lead the work according to the individual’s needs and goals was to adapting co-workers’ working hours and competence to the individual’s needs and goals. Furthermore, another opportunity was that co-workers with the most knowledge of the individual should work most closely to achieve continuity. Another way to work was to have mixed groups of co-workers with different experiences and training to be able to tailor the HC to the individual’s needs and goals. This can be compared to the individual’s owning their daily life and not having to adapt to HC. This follows the principles of human dignity and needs-solidarity and the current value direction to work according to the individual’s needs and goals (National Board of Health and Welfare, 2021b).

The NAs were described as having a close relationship to and knowing how the individual wants HC, which allows them to motivate and challenge the person so that their individual goals could be reached (Tavemark *et al.*, 2023). The FLMs asserted that geographical and relational closeness with the individual, the co-workers and the team was a prerequisite and an opportunity for leading according to the individual’s needs and goals. Overall, this could allow the principles of human dignity and needs-solidarity to prevail.

The FLMs in this study stated that in the top-down system, the organization was an obstacle. This is in line with previous studies that describe that the top-down organization is task-oriented, limiting time and implementing financial control (Hagerman, 2019; Strömberg *et al.*, 2019). The organizational resources are insufficient for elderly care (Hagerman *et al.*, 2019; Kihlgren *et al.*, 2021; Silverglow *et al.*, 2021). In these systems, the cost-effectiveness principle dominated the principles of human dignity and needs-solidarity. This resulted in the work environment being perceived as stressful, which led to increased sick leave. In turn, a poor work environment formed, which negatively affected the individual’s health and HC (Swedish Association of Health Professionals, 2022). The FLMs called for a common leadership style and stated that in the top-down system there was a lack of dialogue and consensus and thereby no mandate to make decisions and lead the work. They described obstacles, such as being “squeezed” in a top-down system in which the politicians rule HC from above. The FLMs must then lead the work with high demands to have a balanced budget without support, which is confirmed by Solbakken *et al.* (2021), Hagerman *et al.* (2015) and Hagerman *et al.* (2019). It is a prerequisite for leading the work that the FLMs have access to organizational support and a continuous dialogue with top management (Hagerman *et al.*, 2019). The struggle can be likened to the FLMs being in two different discourses or systems. One system deals with hiring and scheduling co-workers and keeping the economy in balance (Solbakken *et al.*, 2019). This system can construct their leadership while requiring different forms of approach and giving managers moral problems that challenge their personal values (Aaltvedt *et al.*, 2017).

The FLMs emphasized that there is a lack of leadership style. However, management and leadership are described as two processes: management seeks to produce predictability and order, while leadership produces movement and change (Yukl, 2013). Blom and Alvesson (2015) have stated that the concept of leadership is rarely defined, which may be why no specific leadership style has been adopted in HC. It has been found that most managers are engaged in solving day-to-day problems, managing conflicts between co-workers, keeping costs down, attending meetings and managing administration. Being a manager means managing the organization (Alvesson, 2016; Blom and Alvesson, 2015; Hagerman *et al*, 2019). The organization may have deficiencies in that guidelines and current legislation are not followed (Jarling *et al.*, 2018) as well as deficiencies in medical care and patient safety (Health and Social Care Inspectorate, 2022). These deficiencies may cause the principles of human dignity and needs-solidarity to be set aside.

However, having a clear leadership style in HC is important for health both at the individual level and at the organizational level (Nielsen and Taris, 2019). A prerequisite to lead HC is that the whole organization needs to have the individuals in the centre, and a bottom-up approach is adopted in the organization. HC should be governed according to the individual’s needs and goals and not cost-effectiveness. The bottom-up approach can create a value-based leadership, guided by values and ethical principles that provide a common direction for leadership (Faith, 2013).

Yet, the struggle between conflicting ethical principles can hinder the implementation of structural changes and new ways of working according to the individual’s needs and goals. Critical thinking is needed in which one’s own thinking is questioned and other ways of thinking are considered (Alvesson, 2016).

The living conditions of the older person in HC must involve having the same rights as other people in society.

## Methodological considerations

Because of the Covid-19 pandemic, the data was collected in five HC units, and then the project was cancelled. This results in the exclusion of FLMs from socio-economically vulnerable areas. Trustworthiness has been achieved because we were in the field for two years, and we got to know the stakeholders. In the whole project, data collection methods have been multiple, and the data confirms each other; therefore, the researchers have confidence in the data. In the second interview, we went back to the FLMs so that the data could be confirmed (Lincoln and Guba, 1985; Polit and Beck, 2021). Dependability (Lincoln and Guba, 1985; Polit and Beck, 2021) was also achieved because the same researchers (IJ and ST) did all data collection and have done it through the whole project. Two FLMs were interviewed during the pandemic, which could have affected their experience of leadership. Because of the pandemic, we changed to digital interviews; the managers were used to digital meetings and spoke freely. Digital meetings made it easier for the FLMs to make time and participate in interviews. To enable the reader to determine the transferability of the results to another context (Polit and Beck, 2021), the results have been presented with verbatim quotations.

## Conclusion

The results demonstrate that it is difficult to lead work in HC according to the individual’s needs and goals when FLMs need to struggle between two different systems governing the needs of their role. However, they saw opportunities in leading the work with the individual in the centre, by trusting and seeing co-workers and individuals as valuable human capital. They wanted to facilitate the co-workers to empower the individual in care where working hours and competence needed to be adapted to the individual’s needs and goals. Opportunities needed were to create geographical and relational closeness to primary care, hospital, HC and the individuals. However, the organization’s needs and goals often dominated those of the individual in care when the FLM needs to struggle with the cost-effectiveness principle and maintain a balanced budget.

## Originality/value

The FLMs lack the prerequisites to lead HC according to the individual’s needs and goals. They struggle because they want to have a leadership grounded in the human value and the needs-solidarity principle to lead according to the individual’s needs and goals. To adapt to the cost-effectiveness principle and maintain a balanced budget, they have to assume a management position. There is a need for consensus from the politicians to the co-workers to assume a value-based leadership model based on ethical principles such as the principles of human value and needs-solidarity to lead the HC according to the individual’s needs and goals.

## Figures and Tables

**Table 1.** Participants

Age (years)	No.
31–40	4
41–50	1
51–60	2
61–70	1
*Experience as first-line manager in municipal elderly care (years)*	
0–5	4
6–10	1
11–15	0
16–20	1
21–25	1
>26	1
*FLM in home care service*	*6*
*FLM in home health care*	*2*

**Source:** Authors’ own work
